# A comparative assessment of reference genes in mouse brown adipocyte differentiation and thermogenesis in vitro

**DOI:** 10.1080/21623945.2024.2330355

**Published:** 2024-03-25

**Authors:** Trang Huyen Lai, Jin Seok Hwang, Quang Nhat Ngo, Dong-Kun Lee, Hyun Joon Kim, Deok Ryong Kim

**Affiliations:** a Department of Biochemistry and Convergence Medical Sciences and Institute of Medical Science, Gyeongsang National University, College of Medicine, Jinju, South Korea; b Department of Physiology and Convergence Medical Sciences and Institute of Medical Science, Gyeongsang National University, College of Medicine, Jinju, South Korea; c Department of Anatomy and Convergence Medical Sciences and Institute of Medical Science, Gyeongsang National University, College of Medicine, Jinju, South Korea

**Keywords:** Reference gene, brown adipocytes, Ucp1, thermogenesis, adipocyte differentiation

## Abstract

Adipogenic differentiation and thermogenesis in brown adipose tissue (BAT) undergo dynamic processes, altering phenotypes and gene expressions. Proper reference genes in gene expression analysis are crucial to mitigate experimental variances and ensure PCR efficacy. Unreliable reference genes can lead to erroneous gene expression quantification, resulting in data misinterpretation. This study focused on identifying suitable reference genes for mouse brown adipocyte research, utilizing brown adipocytes from the Ucp1-luciferase ThermoMouse model. Comparative analysis of gene expression data under adipogenesis and thermogenesis conditions was conducted, validating 13 housekeeping genes through various algorithms, including DeltaCq, BestKeeper, geNorm, Normfinder, and RefFinder. *Tbp* and *Rer1* emerged as optimal references for *Ucp1* and *Pparg* expression in brown adipogenesis, while *Tbp* and *Ubc* were ideal for the expression analysis of these target genes in thermogenesis. Conversely, certain conventional references, including *Actb, Tubb5,* and *Gapdh*, proved unstable as reference genes under both conditions. These findings stress the critical consideration of reference gene selection in gene expression analysis within specific biological systems to ensure accurate conclusions.

## 1. Introduction

Brown adipose tissue (BAT) primarily maintains body temperature by generating heat through cold-induced or non-shivering thermogenesis [1,2]. In recent decades, many researchers have focused more on thermogenesis in BAT as it has the potential to enhance energy expenditure, promote the burning of stored fat, and uncover new targets for anti-obesity interventions [3–6]. In BAT, thermogenesis is primarily associated with the activity of uncoupling protein 1 (UCP1), a hallmark protein expressed on the inner mitochondrial membrane of brown adipocytes [7,8]. Many cell and animal models have been developed to study the functions of UCP1 and its modulators. In particular, *Ucp1*-luciferase ThermoMouse, a valuable mouse model, greatly facilitates BAT-related studies on adipogenesis, energy consumption, and thermogenesis [9]. This genetically modified mouse model expresses a luciferase gene under the control of the *Ucp1* gene promoter, enabling researchers to visualize and quantify *Ucp1* expression in real time, either *in vivo* or *in vitro* [9]. Therefore, this mouse model can be effectively utilized for preclinical screening studies and other biological approaches related to adipogenesis and thermogenesis.

UCP1 proteins residing in the mitochondrial inner membrane uncouple the proton gradient potentials used for ATP synthesis in brown adipocytes, leading to an increase in oxygen consumption and heat production, instead of ATP energy [10,11]. In addition to its primary function of UCP1 in thermogenesis, it is also critically associated with browning and adipocyte differentiation, characterized by significant changes in cell morphology and gene expression [12,13]. Additionally, some key transcription factors, including peroxisome proliferator-activated receptor gamma (PPARG) and CCAAT/enhancer-binding protein alpha (CEBPA), play essential roles in adipogenesis [14–16]. These transcription factors induce the expression of numerous genes and trigger distinct morphological and genomic alterations during adipogenesis. In particular, they regulate the formation of lipid droplets and mitochondrial content in differentiated brown adipocytes [10,17].

In gene expression analysis, normalization of mRNA data is of significant importance. This enables a meaningful comparison of mRNA expression levels across various experiments and under different conditions. Several techniques are commonly employed for this normalization, including utilization of internal reference genes (often known as housekeeping genes), total RNA globalization, percentile normalization, and various statistical approaches [18]. The choice of the most suitable method depends on factors such as experimental design, data distribution, and specific goals of the analysis. When utilizing the housekeeping gene method, the relative expression levels of specific genes are typically assessed by comparing them to a reference gene that remains constitutively expressed in cells, thereby providing a valuable biological reference marker. Therefore, using an appropriate reference gene for gene expression studies is critical for defining the cellular or functional characteristics of biological processes. Several cellular housekeeping genes such as *Gapdh, Actb*, and *Tubb5*, consistently expressed inside cells, are widely used as reference genes for expression studies. Nevertheless, it is worth noting that the expression levels of these housekeeping genes can be subject to conditional regulation, resulting in significant variations that depend on factors such as cell type, tissue, developmental stage, and metabolic conditions [19–23]. Unreliable reference genes can mislead the quantification of gene expression, resulting in misinterpretation of data and inaccurate conclusions. Thus, it is crucial to identify suitable housekeeping genes for the development and differentiation of adipose tissue, particularly in the presence of specific experimental variations.

In this study, we evaluated and identified appropriate reference genes for gene expression analysis during brown adipogenesis using two experimental panels: Adipogenesis and Thermogenesis. We selected 13 reference genes (*Actb, B2m, Gapdh, Gusb, Hprt, Pgk1, Rer1, Rpl113a, Rps18, Tbp, Tubb5, Ubc*, and *Sdha*) based on their various cellular functions and evaluated their expression stability using several statistical algorithms. Finally, we identified some suitable genes, such as *Tbp, Rer1, Ubc* which were stably expressed in brown adipogenesis and thermogenesis, and validated their impact on the relative expression of target genes.

## 2. Results

### 2.1. Adipogenesis and thermogenesis using the immortalized stromal vascular fraction (SVF) preadipocytes.

**Figure 1.**
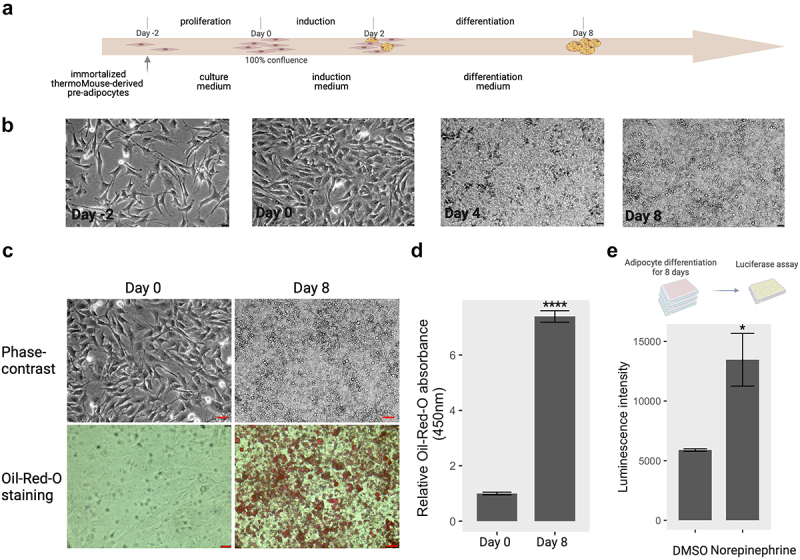
Brown adipocyte differentiation. (a) Induction scheme of brown adipocyte differentiation in vitro from immortalized brown preadipocytes. The details of differentiation process were described under ‘materials and methods’. Created by Biorender.com. (b) Phase-contrast images of cells at day − 2, day 0, day 4, and day 8 during the differentiation process. bar: 50 µm (c) phase-contrast and oil-red-O staining images at pre- adipocytes (day 0) and mature adipocytes (day 8). bar: 100 µm (d) quantification of the relative oil-red-O absorbance between mature adipocytes and preadipocytes. (e) Norepinephrine-induced UCP1 activity on mature adipocytes. Mature adipocytes (day 8) containing Ucp1-luc were incubated with DMSO for control and norepinephrine (1 µM) for 24 hours. The luciferase activity was determined by the method described in materials and methods section. ctr: control; Nore: norepinephrine. * *p* < 0.05, **** *p* < 0.0001.

We first isolated preadipocytes from the interscapular BAT of 3-week-old male Ucp1- luciferase transgenic mice and immortalized brown preadipocytes as described previously [9]. For brown adipogenesis, immortalized preadipocytes were treated with a specific differentiation medium according to the protocol shown in Figure 1(a). Intracellular lipid droplets began to appear in the cytoplasm on day 4 after induction of differentiation, and they were fully accumulated inside cells on day 8, suggesting that brown adipocytes were successfully induced under these conditions (Figure 1(b)). Indeed, based on the quantitative analysis with Oil Red O staining, the intracellular triacylglyceride content of fully differentiated cells (Day 8) was significantly increased compared to that of uninduced cells (Day 0) (Figure 1(c,d)), indicating that these immortalized preadipocytes were successfully differentiated into brown adipocytes.

Norepinephrine (NE), a common thermogenic agent, plays an important role in initiating and maintaining cold-induced non-shivering thermogenesis through the activation of UCP1 in brown adipocytes *in vitro* [24–26]. We tested the luciferase activity of mature brown *Ucp-luc1* adipocytes differentiated for eight days with treatment 1.0 *µ*M NE for 24 h. The luciferase activity in the brown adipocytes responding to NE was significantly upregulated compared to that in non-treated control adipocytes (Figure 1(e)), indicating the successful induction of thermogenesis in mature brown adipocytes with NE treatment.

### 2.2. The q-PCR gene expression analysis of putative reference genes

Based on previous studies, we selected 13 genes with diverse roles in cellular processes. These genes are constitutively and stably expressed in cells. *Actb* (beta-actin) and *Tubb5* (tubulin beta-5 chain) are involved in cellular structural maintenance. *Gapdh* (glyceraldehyde-3-phosphate dehydrogenase), *Sdha* (succinate dehydrogenase complex), and *Pgk1* (phosphoglycerate kinase 1), play key roles in metabolism and energy production. *Rer1* (retention in endoplasmic reticulum 1), *Rpl13a* and *Rps18* (ribosomal proteins) are responsible for protein synthesis. The *Tbp* (TATA box-binding protein) gene is essential for transcription initiation and regulation of gene expression. Additionally, *B2m* (Beta-2-microglobulin), *Gusb (*glucuronidase beta), *Hprt* (hypoxanthine phosphoribosyltransferase), and *Ubc* (ubiquitin C) genes are involved in cellular homoeostasis. These genes were categorized based on their functional annotations from the Reference Sequence (RefSeq) database at NCBI [27], and their primer sequences for amplification and other information are described in Table 1. Primer pairs for the candidate genes were designed using the NCBI Primer-BLAST tool [28].

**Table 1.** Summary of a pair of primers for 13 reference genes and two target genes.

Gene	Gene name (MGI)	NCBI Ref sequence	Primer	Tm (°C)	GC(%)	Amplicon
ActB	Actin, beta	NM_007393.5	F: TTACTGCTCTGGCTCCTAGC	F: 58.88	F: 55.00	**198**
			R: R:CAGCTCAGTAACAGTCCGC	R: 58.26	R: 57.89	
B2m	Beta-2 microglobulin	NM_009735.3	F: TGACCGGCCTGTATGCTATC	F: 59.32	F: 55.00	**115**
			R: GGCGGGTGGAACTGTGTTAC	R: 61.23	R: 60.00	
Gapdh	Glyceraldehyde-3-phosphate dehydrogenase	NM_001289726.2	F: AACCCTTAAGAGGGATGCTGC	F: 60.06	F: 52.38	**120**
			R: CAAATCCGTTCACACCGACC	R: 59.48	R: 55.00	
Gusb	glucuronidase, beta	NM_010368.2	F: TGGCTGGGTGTGGTATGAAC	F: 59.96	F: 55.00	**152**
			R: GGTGACCTCCCTCATGTTCC	R: 59.75	R: 60.00	
Hprt	Hypoxanthine phosphoribosyltransferase	NM_013556.2	F: GAGAGCGTTGGGCTTACCTC	F: 60.46	F: 60.00	**132**
			R: CTAATCACGACGCTGGGACT	R: 59.54	R: 55.00	
Pgk1	Phosphoglycerate kinase 1	NM_008828.3	F: CCATAGCTCCATGGTGGGTG	F: 60.18	F: 60.00	**168**
			R: TTAGCGCCTCCCAAGATAGC	R: 59.61	R: 55.00	
Rer1	Retention in endoplasmic reticulum sorting	NM_026395.2	F: GGAGCTGCGAGTTACAGAATGTC	F: 61.52	F: 52.17	**164**
	receptor 1		R: CCCAGTGTCACAACCCACC	R: 60.53	R: 53.16	
Rpl13a	Ribosomal protein L13A	NM_009438.5	F: CCCACAAGACCAAGAGAGGC	F: 60.32	F: 60.00	**131**
			R: GGTAGGCTTCAGCCGAACAA	R: 60.32	R: 55.00	
Rps18	Ribosomal Protein S18	NM_011296.3	F: AGTTCCAGCACATTTTGCGAG	F: 59.73	F: 47.62	**155**
			R: GAGTTCTCCAGCCCTCTTGG	R: 59.75	R: 60.00	
Tbp	TATA box binding protein	NM_013684.3	F: ACCCTTCACCAATGACTCCTATG	F: 55.00	F: 48.00	**186**
			R: TGACTGCAGCAAATCGCTTGG	R: 54.00	R: 52.00	
Tubb5	Tubulin, beta 5 class1	NM_011655.5	F: GTACCTACCACGGTGACAGC	F:60.11	F: 60.00	**188**
			R: CCCAGACTGACCGAAAACGA	R: 59.97	R: 55.00	
Ubc	Ubiquitin C	NM_019639.4	F: CCCAGTGTTACCACCAAGAAG	F: 58.5	F: 52.38	**103**
			R: CCCATCACACCCAAGAACAAG	R: 59.11	R: 52.38	
Sdha	Succinate dehydrogenase complex, subunit A	NM_023281.1	F: GTCGATGCAGAACCATGCTG	F: 59.62	F: 55.00	**142**
			R: CTCCACCAGGTCTGTGTTCC	R: 59.96	R: 60.00	
Ucp1	Un-coupling Protein 1	NM 009463.3	F: CACCTTCCCGCTGGACACT	F: 55.00	F: 63.00	**91**
			R: CCCTAGGACACCTTTATACCTAATGG	R: 58.00	R: 46.00	
Pparg	Peroxisome Proliferator-Activated Receptor	NM 001127330.3	F: GTGCCAGTTTCGATCCGTAGA	F: 59.00	F: 52.00	**142**
	Gamma		R: GGCCAGCATCGTGTAGATGA	R: 59.00	R: 55.00	

To assess RT-qPCR efficiency, we first performed standard curve analysis on brown adipocytes using day 4 samples (adipocytes on the fourth day after induction of adipogenesis). According to a previous study, this method can assist in avoiding practical and theoretical issues associated with PCR efficiency according to a previous study [29]. RT-qPCR reactions were performed in 10-fold serial dilutions of 500 *n*g cDNA samples obtained from day 4-adipocytes. All PCR reactions exhibited only one single peak for each target gene in the melting curve analysis, suggesting that target-specific amplification resulted in a single and pure amplicon (Figure S1). The PCR efficiency (E) and coefficient of determination (R2) of the target gene primer pairs were determined using the slope of the standard curves (Figure S2). Based on these analyses, all RT-qPCR reactions showed an acceptable value of E (%) and R2, ranging from 80.05 to 97.68 for the efficiency percentage, with associated R2 values of approximately 0.99 (Table 2). These results clearly confirm that the efficiency and specificity of the target gene primer sets used for RT-qPCR amplification in this study were reliable.

**Table 2.** The PCR amplification efficiency of reference and target genes.

No.	Gene	R^2^	Slope	E %
1	Actb	0.9974	−3.7914	83.55018
2	B2m	0.9951	−3.839	82.17321
3	Gapdh	0.9974	−3.8724	81.23321
4	Gusb	0.9854	−3.9038	80.36849
5	Hprt	0.9865	−3.5644	90.78847
6	Pgk1	0.995	−3.8873	80.82062
7	Rer1	0.9889	−3.3788	97.68015
8	Rpl13a	0.9927	−3.8489	81.89237
9	Rps18	0.991	−3.6672	87.36463
10	Tbp	0.9904	−3.6892	86.66439
11	Tubb5	0.9834	−3.8364	82.24727
12	Ubc	0.9977	−3.9781	78.39237
13	Sdha	0.9978	−3.6775	87.03542
14	Ucp	0.9938	−3.9055	80.32219
15	Pparg	0.9962	−3.9157	80.04546

### 2.3. Evaluation of the expression stability of reference gene

To evaluate the expression stability of 13 reference genes in brown adipocytes, we used two experimental models: Adipogenesis and Thermogenesis. For adipogenesis, total RNAs was collected from brown adipocytes on days 0, 2, 4, and 8 of the differentiation process, as shown in Figure 1(a). For thermogenesis, cells on day 0 or day 8 were treated with norepinephrine or DMSO for 24 h to measure the thermogenic effects before total RNAs collection. The candidate reference genes exhibited diverse expression levels in both models, as evident from the the original Ct values obtained through RT-qPCR assays across all experimental conditions (Table S1 and Figure S3). Most candidate genes had mean Ct values between 15 *~* 23. Regardless of the experimental panels, *Gapdh* showed higher Ct values between 28 *~* 30, indicating its lowest expression in the cells. In addition, *Sdha* and *Pgk1* exhibited the highest Ct variations during both adipogenesis and thermogenesis (Table S1).

**Figure 2.**
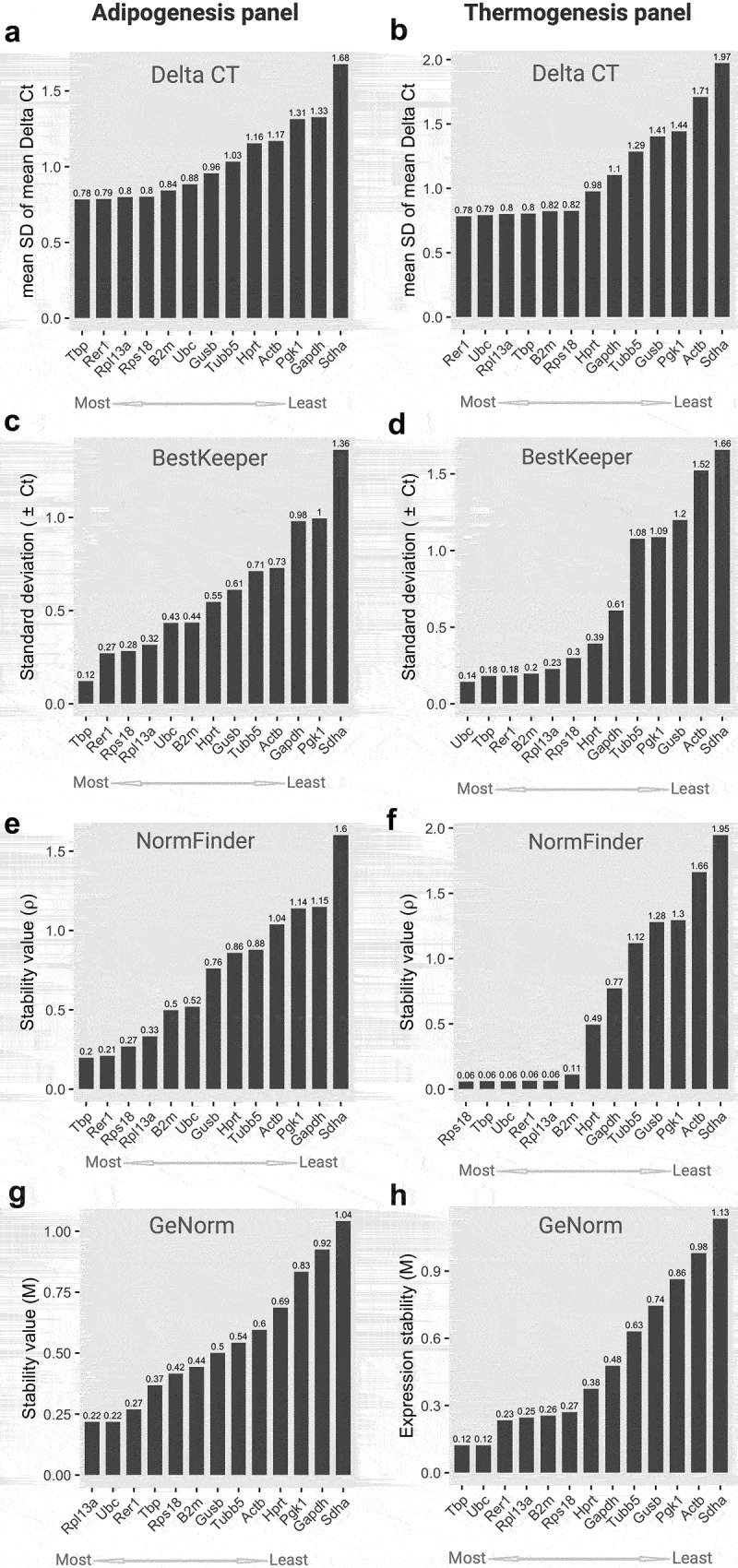
Stability scores and ranking of 13 candidate housekeeping genes in the adipogenesis and thermogenesis panel. (a,b) Stability scores from *Delta CT* algorithms; (c,d) Stability scores from *BestKeeper* algorithms; (e,f) Stability scores from *NormFinder* algorithms; (g,h) Stability scores from *GeNorm* algorithms at an adipogenesis (left) or thermogenesis (right) panel, respectively. For the adipogenesis panel, cells were collected at day 0, day 2, day 4, and day 8 during adipocyte differentiation. For the thermogenesis panel, cells at day 0 and day 8 were treated with norepinephrine (1 *µ*M) or DMSO and incubated for 24 hours. All experiments were performed at three biological replications. The mRNA expression of reference genes underwent examination through RT-qPCR and the RefFinder tool was utilized for calculating stability scores. Original Ct values, including biological replications, from all time points in adipogenesis condition or all treatments in thermogenesis were inputted into the RefFinder tool for analysis. The stability score of individual candidate gene, determined by each algorithm, was displayed as bar-graphs. Lower (left) and higher (right) scores indicate more and less stable genes, respectively.

Next, the gene expression data were subjected to gene stability analysis using four different algorithms (Delta CT, BestKeeper, NormFinder, and GeNorm) integrated with a web-based comprehensive tool, RefFinder.com. The expression stability of each gene was calculated using the original Ct values derived from the two primary models, namely adipogenesis and thermogenesis. The overall stability score of the candidate reference genes was determined by employing the geometric mean rankings from all algorithm scores [30]. The delta CT algorithm was used to compare the differences in Ct values among the reference genes. The average standard deviation (SD) of Ct values for each gene revealed the gene stability score. This indicates that low SD values are more stable [31]. Based on the Delta CT algorithm analysis, *Tbp* exhibited the lowest average SD value (0.78 in the adipogenesis panel, suggesting that it is a most-like reference gene, as shown in Figure 2(a,b). Interestingly, *Rer1* with a mean SD of 0.78, was found to be the most suitable reference gene in the thermogenesis panel. In addition, this gene was identified as one of the best reference candidates for adipogenesis (Figure 2(a,b)). On the contrary, the Delta CT algorithm analysis showed that *Sdha* was the least consistent on both panels with an average SD of 1.68 and 1.97 (Figure 2(a,b)).

Similar to Delta CT, the standard deviation of Ct values was used to rank the reference genes for BestKeeper algorithm analysis. Additionally, we determined the Pearson correlation coefficient (*r*) between reference genes using this algorithm to evaluate their co-regulation. The genes with the lowest SD value and highest r-value were considered to be the most stable, while genes with SD < 1 were believed to be inconsistent [32]. Based on these criteria, *Tbp* (SD = 0.12, *r* = 0.144) and *Rer1* (SD = 0.27, *r* = 0.528) were the most stable genes in the adipogenesis panel, whereas *Ubc* (SD = 0.14, *r* = 0.348), *Tbp* (SD = 0.18, *r* = 0.247), and *Rer1* (SD = 0.18, *r* = 0.907) were ranked as the most stable genes in the thermogenesis panel. In contrast, *Sdha* (SD = 1.36) in the adipogenesis panel and *Sdha* (SD = 1.66), *Actb* (SD = 1.52), *Gusb* (SD = 1.2), *Pgk1* (SD = 1.09), and *Tubb5* (1.08) in the thermogenesis panel exhibited SD > 1, which belong to the least stable genes (Tables S2 and S3) (Figure 2(c,d)).

The NormFinder algorithm was used to determine the stability value (*ρ*) based on the intra- and inter-group variations in candidate reference genes. This algorithm considers both the variation between subgroups and overall variation. As a result, reference genes with lower *ρ* values, especially *ρ* < 0.25, indicate greater stability [33]. We found that *Tbp* (*ρ* = 0.2) and *Rer1* (*ρ* = 0.21) were the most suitable genes for the adipogenesis panel. In contrast, *Actb* (*ρ* = 1.14), *Gapdh* (*ρ* = 1.15), and *Sdha* (*ρ* = 1.6) are unlikely to meet these criteria. In the thermogenesis panel, a group of genes, including *Rps18*, *Tbp*, *Ubc*, *Rer1*, and *Rpl13a* exhibited a *ρ* value of 0.06, suggesting that they are stable reference genes. *Actb* (*ρ* = 1.66) and *Sdha* (*ρ* = 1.95) were at the bottom of the stability value (Figure 2(e,f)).

Finally, we determined the stability values (*M)* of the reference genes using the GeNorm algorithm. They represent the average pairwise variation of a specific control gene with all the other reference genes. That is, the algorithm can identify a combination of two ideal reference genes that are constitutively and stably expressed under experimental conditions. Genes with a lower than 0.5 *M* value generally indicate less variation and greater stability in the gene expression of samples [34]. In the adipogenesis panel, the *M* value between *Rpl13a* and *Ubc* was 0.22, indicating that they were an ideal pair of reference genes. In addition, *Rer1* (*M* = 0.27), *Tbp* (*M* = 0.37), *Rps18* (*M* = 0.42), and *B2m* (*M* = 0.44) exhibited *M* values lower than 0.5. In contrast, *Gapdh* (*M* = 0.92) and *Sdha* (*M* = 1.04) were the least stable genes (Figure 2(g,h)). Interestingly, *Tbp* and *Ubc* (*M* = 0.12) were the most stably expressed genes in the thermogenesis panel, whereas the two least stably expressed genes were *Actb* (*M* = 0.98) and *Sdha* (*M* = 1.13). Other genes, including *Rer1* (*M* = 0.23), *Rpl13a* (*M* = 0.25), *B2m* (*M* = 0.26), *Rps18* (*M* = 0.27), *Hprt* (*M* = 0.38), and *Gapdh* (*M* = 0.48), showed a suitable *M* value, indicating that these genes are stably expressed under thermogenic conditions with less variation.

### 2.4. Overall ranking of the candidate reference genes

**Figure 3.**
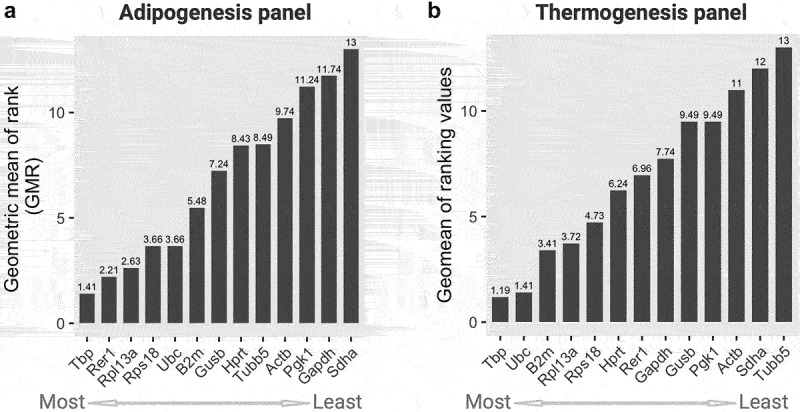
Geometric mean of rankings (GMR) from all four algorithms scores of 13 candidate housekeeping genes in the (a) adipogenesis and (b) thermogenesis panel. The GMR values were calculated by the Refinder algorithm using the original ct values of each gene as input, considering all experimental designs. Lower (left) and higher (right) scores indicate more and less stable genes, respectively.

Next, we applied the RefFinder algorithm to integrate the outputs of Delta CT, Best-Keeper, NormFinder, and GeNorm, and ultimately determine the geometric mean of ranking (GMR), which consequently provides the final evaluation and comprehensive ranking of candidate genes. Lower GMR values indicated more stable gene expression, as suggested previously [30]. In the adipogenesis panel, *Tbp* showed the lowest GMR value (1.41), followed by *Rer1* (GMR = 2.21) (Figure 3(a)). These two genes are the most suitable reference candidates for gene expression analysis during adipogenesis. Some other reference genes, including *Rpl13a*, *Rps18*, and *Ubc* were also reliable, whereas the *Sdha* gene with a GMR value of 13 was the least stable reference gene in the adipogenesis panel. Interestingly, the *Tbp* gene also exhibited the lowest value of GMR (1.19) in the thermogenesis panel, followed by *Ubc* (GMR = 1.41) (Figure 3(b)). Similarly, the *Sdha* (GMR = 12) gene together with *Tubb5* (GMR = 13) were ranked as the least stable genes under these conditions. The gene stability ranking results obtained from the four algorithm-based analyses and a comprehensive RefFinder in adipogenesis and thermogenesis are summarized in Tables 3 and 4, respectively.

**Table 3.** The comparative summary of gene stability for 13 reference genes in the adipogenesis panel.

Method	1	2	3	4	5	6	7	8	9	10	11	12	13
Delta CT	Tbp	Rer1	Rpl13a	Rps18	B2m	Ubc	Gusb	Tubb5	Hprt	Actb	Pgk1	Gapdh	Sdha
BestKeeper	Tbp	Rer1	Rps18	Rpl13a	Ubc	B2m	Hprt	Gusb	Tubb5	Actb	Gapdh	Pgk1	Sdha
Normfinder	Tbp	Rer1	Rps18	Rpl13a	B2m	Ubc	Gusb	Hprt	Tubb5	Actb	Pgk1	Gapdh	Sdha
Genorm	Rpl13a*\*Ubc		Rer1	Tbp	Rps18	B2m	Gusb	Tubb5	Actb	Hprt	Pgk1	Gapdh	Sdha
**Comprehensive ranking**	**Tbp**	**Rer1**	**Rpl13a**	**Rps18**	**Ubc**	**B2m**	**Gusb**	**Hprt**	**Tubb5**	**Actb**	**Pgk1**	**Gapdh**	**Sdha**

Ranking Order 1 => 13 (Better – Good – Average).

**Table 4.** The comparative summary of gene stability for 13 reference genes in the thermogenesis panel.

Method	1	2	3	4	5	6	7	8	9	10	11	12	13
Delta CT	Rer1	Ubc	Rpl13a	Tbp	B2m	Rps18	Hprt	Gapdh	Tubb5	Gusb	Pgk1	Actb	Sdha
BestKeeper	Ubc	Rer1	Tbp	B2m	Rpl13a	Rps18	Hprt	Gapdh	Tubb5	Pgk1	Gusb	Actb	Sdha
Normfinder	Rps18	Tbp	Ubc	Rpl13a	Rer1	B2m	Hprt	Gapdh	Tubb5	Gusb	Pgk1	Actb	Sdha
Genorm	Tbp*\*\Ubc		Rer1	Rpl13a	B2m	Rps18	Hprt	Gapdh	Tubb5	Gusb	Pgk1	Actb	Sdha
**Comprehensive ranking**	**Ubc**	**Tbp**	**Rer1**	**Rps18**	**Rpl13a**	**B2m**	**Hprt**	**Gapdh**	**Tubb5**	**Gusb**	**Pgk1**	**Actb**	**Sdha**

Ranking Order 1 => 13 (Better – Good – Average).

### 2.5. The effect of different reference genes on the expression of target genes

**Figure 4.**
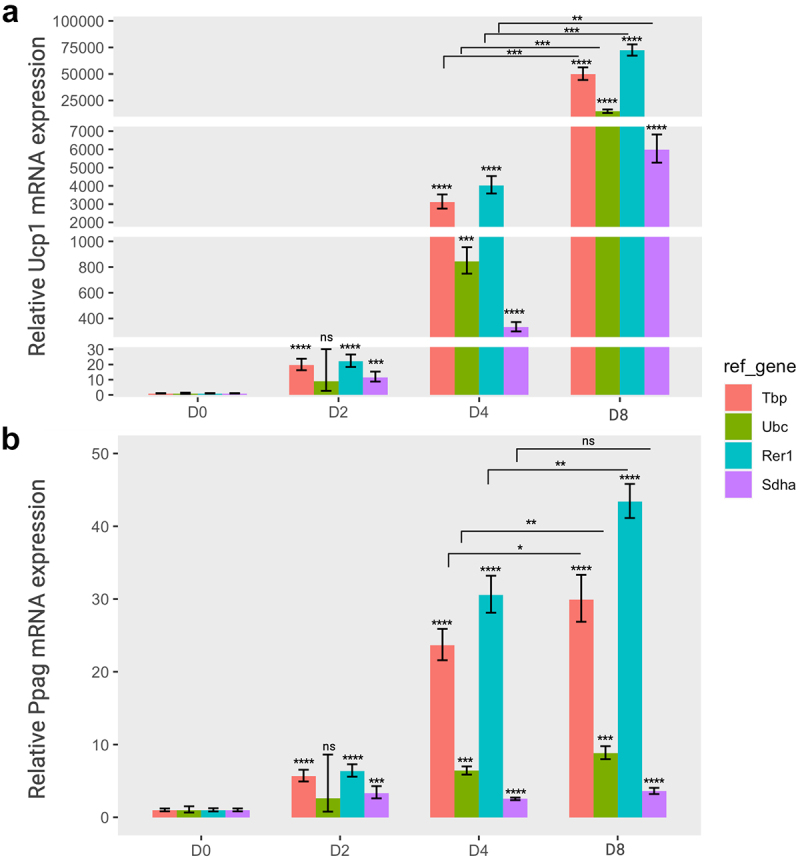
The relative gene expression of adipogenic markers based on four specific reference genes during the brown adipocyte differentiation. The relative expression levels of *Ucp1* (a) and *pparg* (b) normalized to each reference gene (*Tbp*, *Ubc*, *Rer1* and *Sdha*) were represented as graphs. Cells were collected at each differentiation time (day 0 (D0), day 2 (D2), day 4 (D4), and day 8 (D8)), followed by an RT-qPCR assay to assess the mRNA expression of *Ucp1* and *Pparg*. The relative expression of the two target genes was normalized using various reference genes and calculated relative to the control. All experiments were performed at three replications. * *<* 0.05, ** *<* 0.01, *** *<* 0.005, **** *<* 0.001, *ns*: non-significant, *p* value. Orange, green, cyan, and purple represent *Tbp*, *Ubc*, *Rer1* and *Sdha*, respectively.

**Figure 5.**
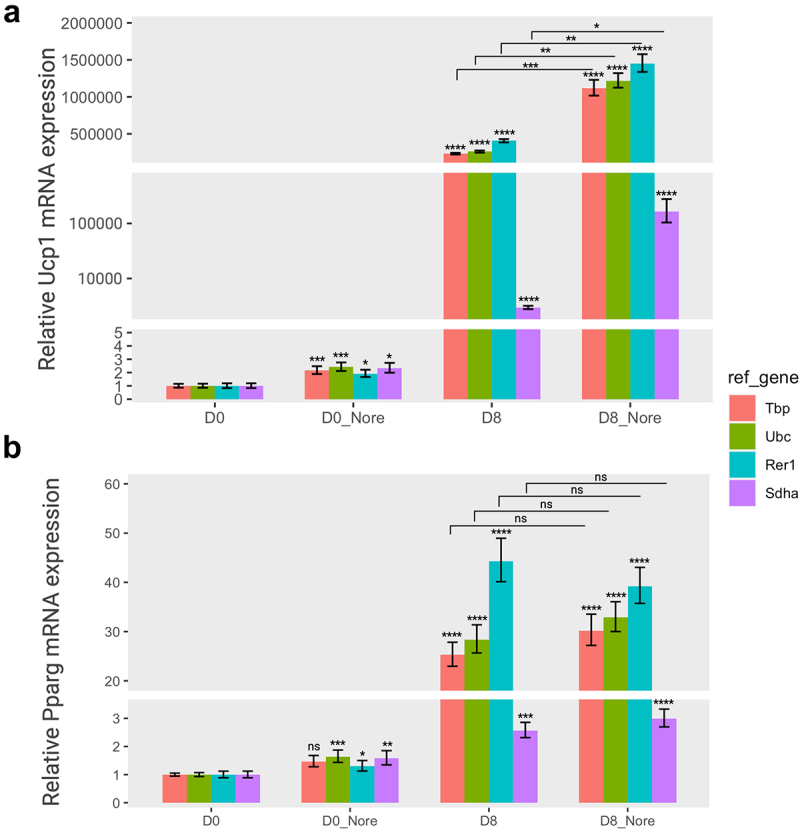
The relative gene expression of adipogenic markers based on four specific reference genes in norepinephrine-induced thermogenesis. The relative expression levels of Ucp1 (a) and pparg (b) normalized to each reference gene (tbp, Ubc, Rer1, and sdha) were represented as graphs. Cells were treated with norepinephrine (1 µM) or DMSO (control) at day 0 (D0, preadipocytes) and day 8 (D8, differentiated adipocytes) and incubated for 24 hours, and subsequently subjected to to RT-qPCR assay to examine the mRNA expression of *Ucp1* and *Pparg*. The relative expression of the two target genes were normalized using various reference genes and calculated in relation to the control. All experiments were performed at three replications. * < 0.05, ** < 0.01, *** < 0.005, **** < 0.001, ns: non-significant. Nore, norepinephrine. Orange, green, cyan, and purple represent *Tbp*, *Ubc*, *Rer1*, and *Sdha*, respectively.

Next, we carried out expression analysis of two target genes, *Pparg* and *Ucp1*, in adipogenesis or thermogenesis, using the selected reference genes. UCP1 proteins are typically expressed at low levels in the early stages of adipogenesis, and its expression in differentiating brown adipocytes gradually increases in a stage-dependent manner. In addition, the *Ucp1* gene is tightly regulated in response to thermogenic stimuli or cold exposure [7,35]. In addition, *Pparg* expression increases during adipogenesis owing to its crucial role in adipocyte differentiation [36,37]. Indeed, *Ucp1* or *Pparg* expression in brown adipocytes significantly increased at the later stages of adipogenesis in a time-dependent manner (Figure 4(a,b)); however, only *Ucp1* expression greatly responded to norepinephrine in brown adipocytes, although the expression of both genes was elevated in differentiated adipocytes (Day 8) regardless of Norepinephrine (Figure 5).

The relative expression levels of these two target genes varied depending on the reference gene. In the adipogenesis panel, the relative expression of *Ucp1* gene was significantly increased until the completion of differentiation (day 8) when normalized to all reference genes. In particular, *Ucp1* expression was higher when using the relatively stable reference genes (*Tbp*, *Rer1*, and *Ubc)* than *Sdha*, which is the most unstable gene (Figure 4(a)). The expression of *Pparg* exhibited a similar pattern for both *Tbp* and *Rer1* reference genes. However, the relative *Pparg* expression at the differentiation stage of brown adipocytes was very low when either *Ubc* or *Sdha* was used as a reference gene (Figure 4(b)). Indeed, the *Ubc* gene was less stable in the adipogenesis panel, although it was ranked as the second most stable gene in thermogenesis (Figure 3).

Similarly, the relative expression of the *Ucp1* gene was greatly increased in mature adipocytes (day 8) compared to uninduced preadipocytes (day 0) when normalized to *Tbp*, *Rer1*, and *Ubc*. In addition, the *Ucp1* relative levels were notably increased upon treatment with norepinephrine in differentiated adipocytes (day 8) in the thermogenesis panel (Figure 5(a)). In addition, norepinephrine activated UCP1 expression in uninduced brown preadipocytes (day 0). In the same panel, the relative *Pparg* expression was consistently increased in differentiated adipocytes (day 8) compared to that in preadipocytes (day 0) but did not respond to norepinephrine, as expected (Figure 5(b)). All selected reference genes (*Tbp*, *Rer1*, and *Ubc*), except *Sdha* showed a similar effect on *Pparg* expression. The relative expression levels of the two target genes revealed a striking contrast when normalized to the most stable versus least stable genes, suggesting that a specific reference gene might considerably impact the assessment of gene expression under various experimental conditions.

## 3. Discussion

Brown adipocytes, which are specialized fat cells, can generate heat, which regulates body temperature and energy expenditure [2,17]. Therefore, understanding the molecular mechanisms behind these events is essential to evaluate the physiological significance and potential therapeutic implications of brown adipose tissues in metabolic disorders and obesity-related conditions. Many studies using mouse models and brown adipogenesis suggest that they could be utilized to develop a new potential therapy by regulating adipose tissue browning and thermogenic activity [9,38,39]. In this study, we generated immortalized brown preadipocytes isolated from the interscapular BAT of Ucp1-luciferase transgenic mice and performed gene expression analysis during brown adipogenesis in vitro. To screen for new therapeutic targets in adipogenesis, we first examined possible reference genes that could be used for normalization of gene expression. In particular, selecting a suitable reference is critical for studying the function of genes that are differentially expressed during the developmental stages of adipocytes. Indeed, according to a previous study [19], some reference genes showed different gene expression stabilities in the adipose tissues of mice with metabolic disorders based on real-time PCR analysis.

In diverse scientific disciplines, there has been a notable increase in the number of studies validating reference genes for RT-qPCR normalization. Different methodologies employ varied approaches to rank these genes, and consequently, relying on a single method may not yield the definitive ‘best’ reference gene. To address this, multiple approaches are often employed to ascertain the most suitable reference gene. In our study, we utilized the RefFinder tool [30], which integrates four contemporary computational programmes (geNorm [34], NormFinder [33], BestKeeper [32], and the comparative ΔCt method [21]. This comprehensive approach enabled the assessment of stability and reliability of 13 well-established reference genes, each serving distinct functions (as detailed in Table 1), across two *in vitro* experimental conditions: adipogenesis and thermogenesis. A similar strategy has been widely adopted to evaluate the expression stability of reference genes under various experimental conditions [21–23,40].

Among the 13 candidate genes, we identified two genes (*Tbp* and *Rer1*) as the optimal references for gene expression analysis of target genes in brown adipogenesis and thermogenesis (Figure 3). These results are consistent with several previous studies in which *Tbp* was stably and consistently expressed across adipose tissues, including white adipose tissue (WAT) and brown adipose tissue (BAT) [19,20]. Additionally, the *Tbp* gene has been suggested as a reference for qPCR-based gene expression analyses in peripheral nerve injury conditions [41], liver cell-injured models [42], osteogenic differentiation [43], and in the ageing models [44]. Since the TBP protein plays an essential role in transcription initiation to maintain cellular homoeostasis, its expression should be stable during cell differentiation. Indeed, TBP, as a general transcription factor, directly binds to the TATA box sequence of promoters in most genes and serves as a crucial component of the general transcription machinery [45]. In addition, the *Rer1* (Retention in endoplasmic reticulum 1) gene exhibited remarkable stability across the two experimental conditions, consistently normalizing the expression of target genes, *Ucp1* and *Pparg* (Figures 4 and 5). Although *Rer1* is not conventionally employed as a reference gene in adipocyte research, in our specific experimental context, it demonstrated stability and suitability as one of the most reliable reference genes for expression analysis in mouse brown adipocytes. Generally, Rer1 is recognized as a well-conserved early Golgi membrane protein crucial for cellular homoeostasis and maintaining the functional integrity of the endoplasmic reticulum (ER) [46]. A study by Taichi Hara *et. al* showed that Rer1 is essential for neural stem cell maintenance, and its depletion can lead to malformation in the mouse cerebral cortex [47]. They also suggested that the quality control systems in the late-Golgi or post-Golgi stages exhibit a more stringent function in mouse cells compared to other cultured cell lines, offering a potential explanation for the observed stability of *Rer1* gene in our experimental context.

Moreover, we identified that the most traditional reference genes belonging to two groups, cellular structural maintenance (*Actb* and *Tubb5)* and metabolism and energy production (*Gapdh*, *Pgk1*, and *Sdha*), displayed expression variability in our experimental conditions, securing mid to low rankings among the 13 candidate genes. *Actb* and *Gapdh* are commonly used as endogenous control genes to assess relative gene expression owing to their high abundance. However, in this study, the *Actb* gene was ranked only 10^th^ in the adipogenesis panel and 11^th^ in the thermogenesis panel, while the *Gapdh* gene was identified as a less stable gene, ranking 12^th^ in the adipogenic panel and 8^th^ in the thermogenic panels. Our findings align with numerous studies, demonstrating that *Gapdh* and *Actb*, housekeeping genes frequently mentioned in the literature, may not be optimal choices for our experimental design. Their gene expression levels were relatively unstable in various contexts, including the mouse choroid plexus [20], T lymphocytes co-cultured with mesenchymal stem cells [40], ageing studies [44], and human adipose stromal cells [48]. This suggests that these reference genes might be specifically modulated during adipogenesis and thermogenesis, which consequently induces structural and metabolic alterations in brown adipocytes. In general, cellular metabolic activities specifically respond to external signals at different developmental stages, indicating relatively unstable expression patterns of genes associated with cellular structure and metabolism, as shown in the study. Using the least stable gene, *Sdha* to normalize the target genes revealed a substantial difference in the relative expression levels of *Ucp1* and *Pparg*. This was unexpectedly lower than the consistent findings obtained for the most stable genes, *Tbp1* and *Rer1* (Figures 4 and 5).

In summary, our findings suggest that *Tbp* and *Rer1* present promising options as reference genes for RT-qPCR-based gene expression analysis in the context of adipogenesis and thermogenesis, specifically in mouse brown adipocytes under in vitro conditions. Nevertheless, it is crucial to acknowledge that our analysis concentrated on a specific set of candidate reference genes within mouse cells and in vitro experimental settings, potentially limit their generalizability. Furthermore, aligning with prior research, we strongly advocate for the implementation of validation experiments to identify suitable reference genes before delving into the study of specific cellular functions.

## 4. Materials and methods

### 4.1. Reagents and drugs

Reagents and drugs used in this study were listed as follows: Dulbecco Modified Eagle Medium (DMEM) (high glucose, #11965092), DMEM/F-12 (HEPES, #11330032), foetal bovine serum (FBS; #16000–044), RNase-free (#89836), DNase I Solution (1 unit/*µ*L), and RevertAid First Strand cDNA Synthesis Kit from Thermo Scientific; 3-Isobutyl-1-Methylxanthine (IBMX, #I5879), Indomethacin (#I7378), Dexametha- sone (#D4902), Insulin from porcine pancreas (#I5523), 3,3,5-Triiodo-L-thyronine (#T2877), Norepinephrine (#N5785), Collagenase D (#COLLD-RO), Dispase II (#42613-33-2) from Sigma Aldrich; Trizol reagent (#15596026), PureLink Genomic DNA Mini Kit from Invitrogen; amfiSure qGreen Q-PCR Master Mix(2X), With- out ROX (#Q5600–005) from GenDEPOT. G418 (#10131–035) and Lipofectamine 3000 (#11668–500) from Gibco (Grand, Island NYUSA). Protease inhibitor cocktails (#78441), Enhanced ChemiLuminescence (ECL) detection system (#34080), and M2 lysis buffer (#85111) were purchased from Thermo Scientific.

### 4.2. Generation of immortalized UCP1 luciferase preadipocyte lines

The stromal vascular fraction (SVF) was isolated from both sides of the interscapular BAT of a 3-week-old male ThermoMouse mouse strain (JAX stock #026690) euthanized with an intraperitoneal injection of avertin (2,2,2 Tribromoethanol, 250 mg/kg). Animal experiments followed the National Institute of Health (NIH) guidelines and a scientifically reviewed protocol (GLA-100917-M0093). The Animal Care Committee for Animal Research at Gyeongsang National University approved the study protocol (GNU-200820-M0053). The study was conducted according to the ARRIVE guidelines 2.0 (https://arriveguidelines.org) [49]. The cells isolated from the SVF were then cultured in Dulbecco’s modified Eagle’s medium (DMEM) with 10% FBS and immortalized using the retrovirus-mediated simian virus 40 large T antigen (SV40 LT), as described previously [50]. Immortalized preadipocytes were selected in media containing puromycin (2 *µ*g/*m*l) and verified by genotyping following the manufacturer’s protocols (Jackson Laboratory).

### 4.3. Cell culture and brown adipocyte differentiation

The immortalized preadipocytes were maintained in standard high-glucose DMEM supplemented with 10% FBS and 100 µg/mL streptomycin and incubated in a 37^*o*^C humidified atmosphere containing 5% CO2. For the brown adipocyte differentiation, immortalized preadipocytes were seeded at an initial cell concentration of 1 × 10[5] cells/ml and incubated until cell confluency reached 90% − 100%. The cells were then cultured in an induction medium (DMEM/F-12 medium, 10% FBS, 0.5 *m*M Isobutylmethylxathin (IBMX), 0.125 *m*M Indomethacin, 1.0 *µ*M Dexamethasone, 5.0 *µ*g/ml insulin, 1.0 nM T3) for two days, and subsequently maintained for six consecutive days in differentiation medium (DMEM/F-12 medium, 10% FBS, 5.0 *µ*g/ml insulin, 1.0 *n*M T3).

### 4.4. Cell culture and brown adipocyte differentiation

The immortalized preadipocytes were maintained in standard high-glucose DMEM supplemented with 10% FBS and 100 µg/mL streptomycin and incubated in a 37°C humidified atmosphere containing 5% CO2. For the brown adipocyte differentiation, immortalized preadipocytes were seeded at an initial 1 × 10[5] cells/ml cell concentration and incubated until cell confluency reached 90% − 100%. The cells were then cultured in an induction medium (DMEM/F-12 medium, 10% FBS, 0.5 mM Isobutylmethylxathin (IBMX), 0.125 mM Indomethacin, 1.0 µM Dexamethasone, 5.0 µg/ml insulin, 1.0 nM T3) for two days, and subsequently maintained for six consecutive days in differentiation medium (DMEM/F-12 medium, 10% FBS, 5.0 µg/ml insulin, 1.0 nM T3).

### 4.5. Oil red O staining

The Oil Red O (ORO) solution was freshly prepared by mixing Oil Red O stock solution (0.03% Oil Red O in 100% isopropanol) in a mixture of 60:40 stock solution with distilled water (DW). The cells were fixed in a 4% formaldehyde solution for 30 min at room temperature and then washed with DW three times. Subsequently, 60% isopropanol was added to the cells, allowed to sit for five minutes, and removed without washing. The cells were evenly covered with ORO solution, the cell plates were rotated, and incubated at room temperature for 10–20 min. After incubation, the ORO solution was removed, and the cells were gently washed with two to five DW washes until no excess dye solution was visible. The cells were then submerged in DW to avoid drying, and examined under a bright-field microscope within 24 h before quantitative analysis. To quantify the ORO staining assay, the stained cells were extracted in 100% isopropanol with a 50% volume of the cell plate (e.g. for a 24-well plate, 250 *µ*l per well), and 80% of the extraction volume was used to measure the absorbance at a wavelength of 492 *n*m in a microplate reader. Isopropanol (100%) was used as the background control to subtract the background signal.

### 4.6. Primer design

Primers were designed using Primer-BLAST with a targeted amplicon product size of 70–200 bp in length, a G-C content of 50–60%, and a melting temperature (Tm) aiming for a minimum of 60^*o*^C and a maximum of 63^*o*^C. Primers of the reference genes and target genes used in the assay are listed in Table 1, with detailed information on the designed primers used in this study, including NCBI reference sequences, primer sequences, G-C content, and Tm.

### 4.7. RNA extraction and RT-qPCR

The cells were collected at the indicated times and used for RNA extraction according to the manufacturer’s instructions. Briefly, total RNAs from six-well plates was extracted with 1.0 *m*L Trizol for each well. For the matured adipocyte samples, additional centrifugation of the Trizol-containing cell lysate was performed at 12,000 rpm for 10 min to remove excess fat. We verified that the A260/A280 ratios were between 1.8 and 2.0. Before cDNA synthesis, 1.0 *µ*g of total RNA sample was treated with 1 U DNase I solution for 30 min at 37^*o*^C °C to remove trace amounts of DNA. Next, a Thermo Scientific RevertAid First Strand cDNA Synthesis Kit was used to generate first-strand cDNA using RNA templates for reverse transcription, according to the manufacturer’s instructions. Subsequently, qPCR was performed using the amfiSure qGreen Q-PCR Master Mix kit following the manufacturer’s protocol with 100 *n*g cDNA synthesis products in a 10 *µ*l reaction. Reactions were performed using Rotor-Gene Q (QIAGEN, Hilden, Germany).

Target gene expression was quantified in the differentiated samples relative to the selected reference genes relative to the control sample (day 0). The ∆∆*C*_*t*_ model was applied using Pearson pcr R package [51]. The relative expression of the target genes was compared on each differentiation day to the control cells (day 0) using Student’s *t*-test. *p*-values *<* 0.05. Experiments were performed in triplicate.

### 4.8. RT-qPCR standard curve

The RT-qPCR standard curve method was performed using SVFs four days (day 4) after the induction of adipogenesis with six data points over several orders of 10-fold dilutions. An RT-qPCR standard curve was graphically represented as a semi-log regression line plot of the CT values versus the log of each sample dilution. The R^2^ value is the coefficient of correlation obtained for the standard curve and should be *>* 0.99, to provide good confidence within the correlation. Efficiency (*E*%) was calculated from *slope* of the standard curve according to the following equation:

$$\mathrm{E\%}=\;\left[\frac{-1}{10^{\mathrm{slope}}-1}\right]*100\%$$

### 4.9. Stability score analysis

The stability of gene expression was examined using RefFinder, a web-based comprehensive tool [52] created for the analysis and screening of reference genes, to compare and rank the evaluated potential reference genes. The tool incorporates four key algorithms NormFinder [33], GeNorm [34], BestKeeper [32], and Delta CT [21]. To employ the RefFinder tool, we inputted the original Ct values, encompassing all biological replications at different time points for adipogenesis conditions or treatments for thermogenesis conditions for each gene, adhering to the instructions provided on the RefFinder platform [30]. The output stability scores from RefFinder included NormFinder, *ρ*; GeNorm, *M*; BestKeeper, *r*; Delta CT: Mean SD of mean *~*Ct; and comprehensive gene stability RefFinder: Geomean. The geometric means of the ranks were calculated from all four algorithms and provided a comprehensive final ranking of the candidate reference genes.

### 4.10. Software environment and reproducibility

R and Bioconductor programmes were used to perform the relative expression of target genes, statistical analysis, and data visualization [53–55].

### 4.11. Statistical analysis

For each sample, RT-qPCR was independently performed at least three times. Data are presented as mean values (*±* S.D). The normal distribution of the Ct values derived from the RT-qPCR assays was determined by Shapiro-Wilk test [56]. The statistical significance of the two groups was evaluated using an unpaired Student’s *t*-test, in the **t.test()** function conducted in R (Vienna, Austria) [53]. *p <* 0.05.

## Supplementary Material

Supplemental Material

## Data Availability

The reference sequences used for designing primers in this study can be found in the NCBI Reference Sequences (NM007393.5; NM009735.3; NM001289726.2; NM010368.2; NM013556.2; NM008828.3; NM026395.2; NM009438.5; NM011296.3; NM013684.3; NM011655.5; NM019639.4; NM023281.1; NM009463.3; NM001127330.3).

## Accession codes

The code used to generate the figures and tables and run the analysis in this study is open-source (GPL-3) and freely available at https://github.com/BCMSLab.
