# Supplementary material for: A comparative assessment of reference genes in mouse brown adipocyte differentiation and thermogenesis in vitro
Source: Adipocyte. 2024 Mar 25;13(1):2330355. doi: 10.1080/21623945.2024.2330355 (PMC10965104; doi:10.1080/21623945.2024.2330355)
Supplement: Supplemental Material [file KADI_A_2330355_SM0715.docx]

**Supplementary Information for:**

A comparative assessment of reference genes in brown adipocyte differentiation and thermogenesis

**Trang Huyen Lai, Jin Seok Hwang, Quang Nhat Ngo, Dong-Kun Lee, Hyun Joon Kim and**

**Deok Ryong Kim**

**
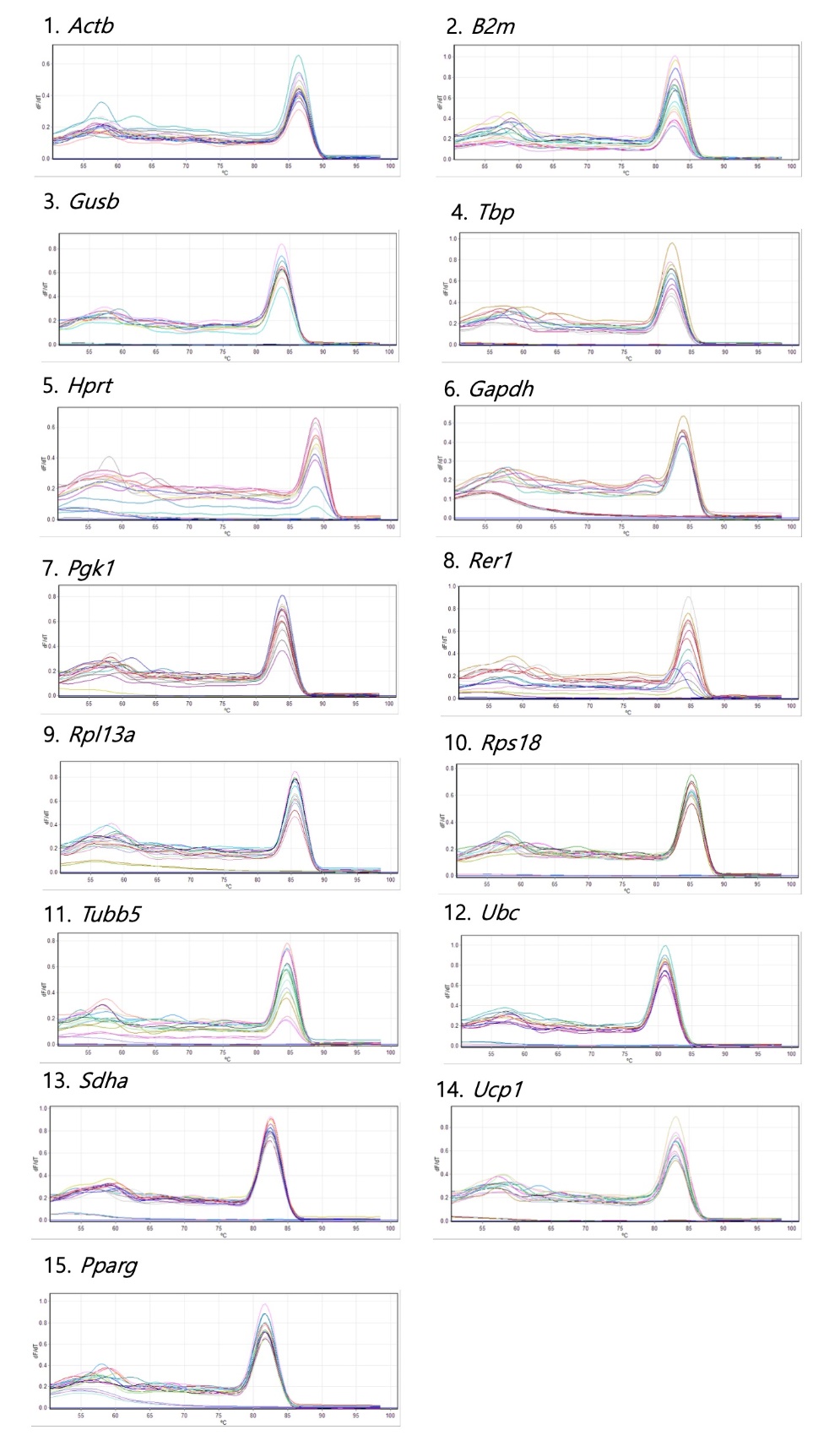
**

**Figure S1:** Melting curves analysis.

**
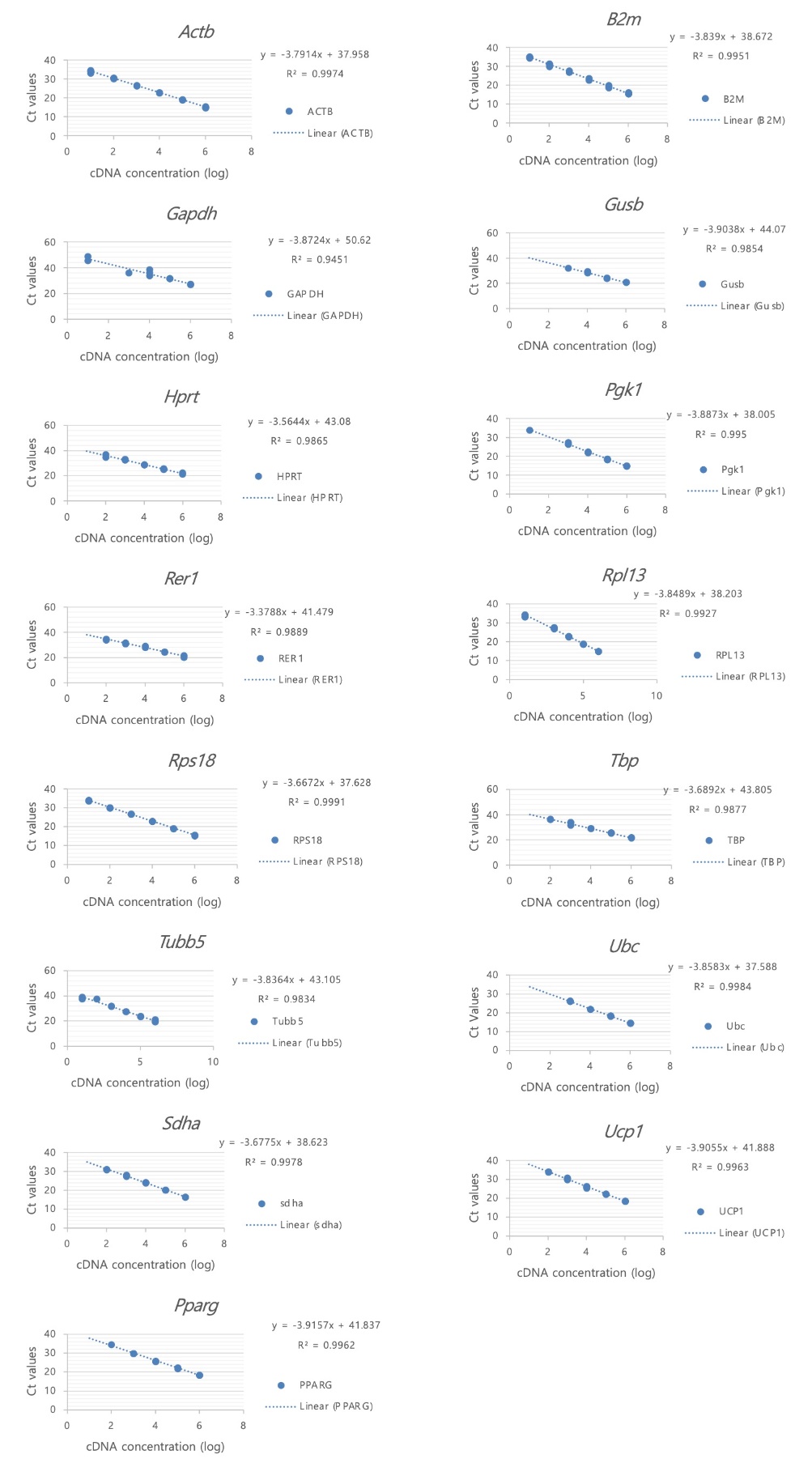
**

**Figure S2:** Standard curve analysis of primers for the candidate housekeeping genes.

**
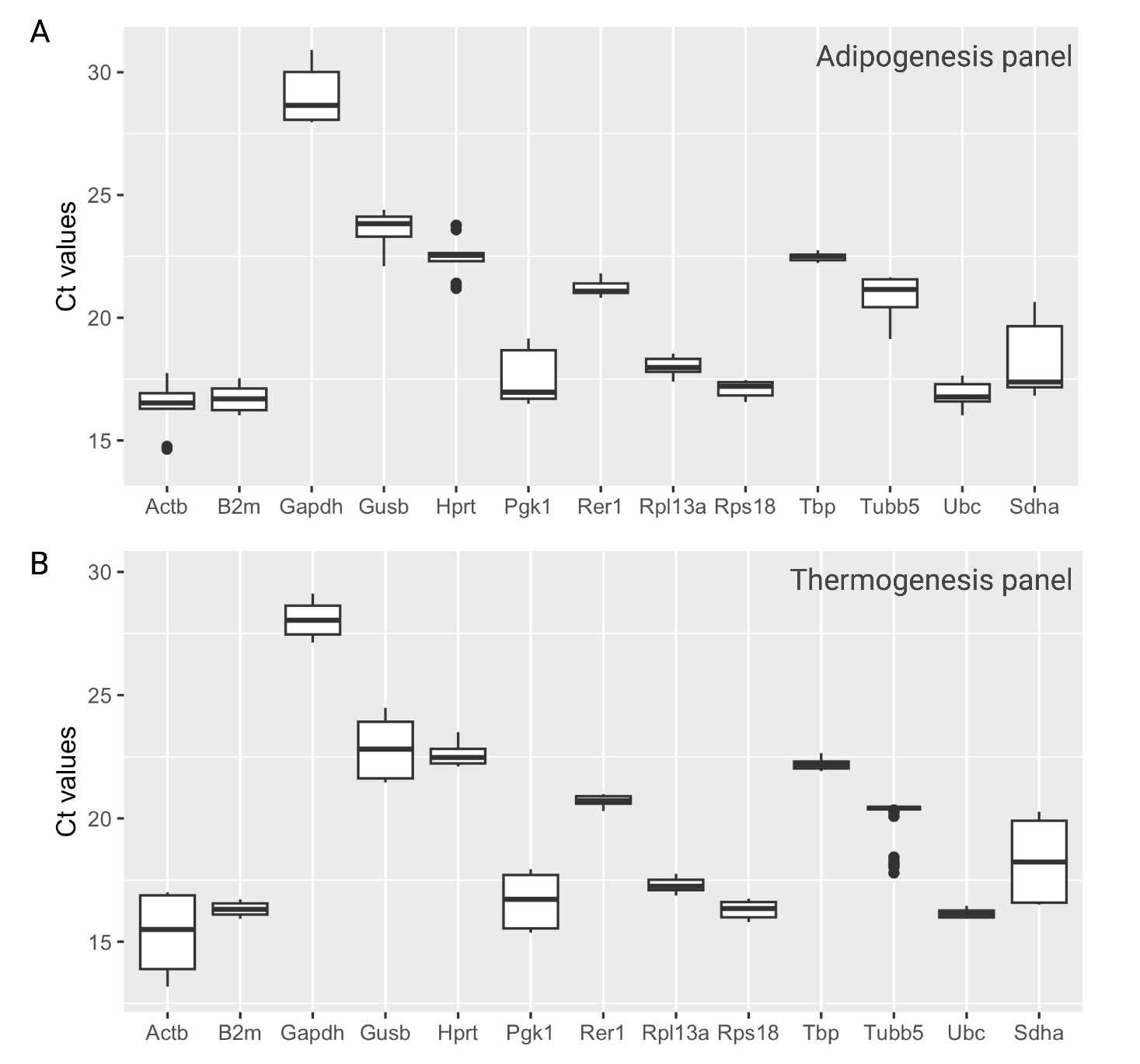
**

**Figure S3**: RT-qPCR cycle threshold (Ct) values for the 13 candidate reference genes were assessed across samples in (A) Adipogenesis panel (Brown-adipocyte differentiation), encompassing cells collected at day 0 (D0), day 2 (D2), day 4 (D4), day 6 (D6), and day 8 (D8) after induction of differentiation. Similarly, (B) Thermogenesis panel (norepinephrine-induced thermogenesis) involved samples collected at day 0 and day 8 with/without Norepinephrine-treatment. Candidate housekeeping genes include *Actb*, *B2m*, *Gapdh*, *Gusb*, *Hprt*, *Pgk1*, *Rer1*, *Rpl13a*, *Rps18*, *Tbp*, *Tubb5*, *Ubc*, *Sdha*.

Table S1: Mean and SD of Ct values of 13 reference genes in adipogenesis and thermogenesis models.

| Gene | Adipogenesis | | Thermogenesis | |
| --- | --- | --- | --- | --- |
|  | Mean Ct | SD | Mean Ct | SD |
| *Actb* | 16.40 | ± 0.97 | 15.30 | ± 1.56 |
| *B2m* | 16.69 | ± 0.49 | 16.33 | ± 0.24 |
| *Gapdh* | 29.06 | ± 1.05 | 28.07 | ± 0.65 |
| *Gust* | 23.57 | ± 0.73 | 22.85 | ± 1.22 |
| *Hprt* | 22.49 | ± 0.77 | 22.61 | ± 0.47 |
| *Pgk1* | 17.55 | ± 1.04 | 16.66 | ± 1.10 |
| *Rer1* | 21.23 | ± 0.31 | 20.72 | ± 0.21 |
| *Rpl13a* | 17.99 | ± 0.37 | 17.28 | ± 0.26 |
| *Rps18* | 17.11 | ± 0.33 | 16.33 | ± 0.33 |
| *Tbp* | 22.47 | ± 0.14 | 22.20 | ± 0.21 |
| *Tubb5* | 20.82 | ± 0.83 | 19.21 | ± 1.09 |
| *Ubc* | 16.87 | ± 0.52 | 16.16 | ± 0.17 |
| *Sdha* | 18.35 | ± 1.43 | 18.27 | ± 1.66 |

Table S2: Analyzing stability scores of housekeeping genes by BestKeeper algorithm on adipogenesis panel.

|  | *Actb* | *B2m* | *Gapdh* | *Gusb* | *Hprt* | *Pgk1* | *Rer1* | *Rpl13a* | *Rps18* | *Tbp* | *Tubb5* | *Ubc* | *Sdha* |
| --- | --- | --- | --- | --- | --- | --- | --- | --- | --- | --- | --- | --- | --- |
| n | 15 | 15 | 15 | 15 | 15 | 15 | 15 | 15 | 15 | 15 | 15 | 15 | 15 |
| geo Mean (Ct) | 16.37 | 16.69 | 29.04 | 23.56 | 22.48 | 17.52 | 21.23 | 17.99 | 17.11 | 22.47 | 20.8 | 16.86 | 18.29 |
| AR Mean (Ct) | 16.4 | 16.69 | 29.06 | 23.57 | 22.49 | 17.55 | 21.23 | 17.99 | 17.11 | 22.47 | 20.82 | 16.87 | 18.35 |
| min (Ct) | 14.64 | 16.02 | 27.96 | 22.1 | 21.19 | 16.49 | 20.81 | 17.4 | 16.56 | 22.23 | 19.13 | 16.02 | 16.82 |
| max (Ct) | 17.75 | 17.53 | 30.91 | 24.4 | 23.77 | 19.15 | 21.8 | 18.54 | 17.46 | 22.75 | 21.65 | 17.64 | 20.64 |
| **std dev (***±* **Ct)** | **0.73** | **0.44** | **0.98** | **0.61** | **0.55** | **0.99** | **0.27** | **0.32** | **0.28** | **0.12** | **0.71** | **0.43** | **1.36** |
| CV (% Ct) | 4.45 | 2.61 | 3.37 | 2.6 | 2.44 | 5.67 | 1.27 | 1.75 | 1.65 | 0.54 | 3.42 | 2.57 | 7.42 |
| min (x-fold) | -3.33 | -1.59 | -2.11 | -2.75 | -2.44 | -2.04 | -1.34 | -1.5 | -1.46 | -1.18 | -3.18 | -1.8 | -2.78 |
| max (x-fold) | 2.59 | 1.79 | 3.66 | 1.79 | 2.45 | 3.1 | 1.49 | 1.46 | 1.28 | 1.22 | 1.8 | 1.71 | 5.09 |
| std dev (*±* x-fold) | 1.66 | 1.35 | 1.97 | 1.53 | 1.46 | 1.99 | 1.21 | 1.24 | 1.22 | 1.09 | 1.64 | 1.35 | 2.57 |
| coeff. of corr. (r) | 0.664 | 0.412 | 0.001 | 0.703 | 0.001 | 0.001 | 0.528 | 0.296 | 0.455 | 0.144 | 0.779 | 0.289 | 0.001 |
| p-value | 0.007 | 0.127 | 0.246 | 0.003 | 0.064 | 0.145 | 0.043 | 0.285 | 0.089 | 0.609 | 0.001 | 0.297 | 0.022 |

Table S3: Analyzing stability scores of housekeeping genes by BestKeeper algorithm on thermogenesis panel.

|  | *Actb* | *B2m* | *Gapdh* | *Gusb* | *Hprt* | *Pgk1* | *Rer1* | *Rpl13a* | *Rps18* | *Tbp* | *Tubb5* | *Ubc* | *Sdha* |
| --- | --- | --- | --- | --- | --- | --- | --- | --- | --- | --- | --- | --- | --- |
| n | 12 | 12 | 12 | 12 | 12 | 12 | 12 | 12 | 12 | 12 | 12 | 12 | 12 |
| geo Mean (Ct) | 15.22 | 16.33 | 28.06 | 22.82 | 22.6 | 16.63 | 20.72 | 17.28 | 16.32 | 22.2 | 19.18 | 16.16 | 18.2 |
| AR Mean (Ct) | 15.3 | 16.33 | 28.07 | 22.85 | 22.61 | 16.66 | 20.72 | 17.28 | 16.33 | 22.2 | 19.21 | 16.16 | 18.27 |
| min (Ct) | 13.18 | 15.94 | 27.13 | 21.46 | 22.11 | 15.37 | 20.3 | 16.87 | 15.8 | 21.92 | 17.79 | 15.93 | 16.5 |
| max (Ct) | 17.01 | 16.72 | 29.12 | 24.49 | 23.5 | 17.94 | 20.99 | 17.76 | 16.75 | 22.65 | 20.42 | 16.46 | 20.28 |
| **std dev (***±* **Ct)** | **1.52** | **0.2** | **0.61** | **1.2** | **0.39** | **1.09** | **0.18** | **0.23** | **0.3** | **0.18** | **1.08** | **0.14** | **1.66** |
| CV (% Ct) | 9.95 | 1.21 | 2.17 | 5.25 | 1.73 | 6.53 | 0.87 | 1.31 | 1.84 | 0.81 | 5.6 | 0.88 | 9.06 |
| min (x-fold) | -4.12 | -1.31 | -1.91 | -2.56 | -1.41 | -2.39 | -1.34 | -1.33 | -1.44 | -1.21 | -2.62 | -1.17 | -3.24 |
| max (x-fold) | 3.45 | 1.31 | 2.08 | 3.19 | 1.86 | 2.48 | 1.21 | 1.39 | 1.35 | 1.37 | 2.36 | 1.23 | 4.24 |
| std dev (*±* x-fold) | 2.87 | 1.15 | 1.52 | 2.3 | 1.31 | 2.13 | 1.13 | 1.17 | 1.23 | 1.13 | 2.11 | 1.1 | 3.15 |
| coeff. of corr. (r) | 0.817 | 0.225 | 0.001 | 0.727 | 0.001 | 0.001 | 0.907 | 0.809 | 0.859 | 0.250 | 0.779 | 0.348 | 0.001 |
| p-value | 0.001 | 0.481 | 0.007 | 0.007 | 0.397 | 0.019 | 0.001 | 0.001 | 0.001 | 0.433 | 0.003 | 0.267 | 0.009 |
